# Post-Cholecystectomy Syndrome

**DOI:** 10.5811/cpcem.2017.6.33321

**Published:** 2017-11-03

**Authors:** Tami Moore, Manish Amin

**Affiliations:** Kern Medical, Department of Emergency Medicine, Bakersfield, California

## CASE PRESENTATION

A 32-year-old female with a history of cholecystectomy three years prior, presented to the emergency department with epigastric pain. Liver function tests (LFTs) were abnormal (total bilirubin: 1.4mg/dl, alkaline phosphatase: 117U/L, aspartate transaminase: 294U/L, alanine transaminase: 189U/L), however ultrasound (US) imaging was negative for gallbladder pathology and the patient was discharged home with normal vital signs and instructed to follow up in two days if symptoms persisted. At her follow up visit, her LFTs worsened (total bilirubin: 3.1mg/dl, alkaline phosphatase: 172U/L, aspartate transaminase: 230U/L, alanine transaminase: 518U/L) and the patient underwent a magnetic resonance cholangiopancreatography (MRCP) which showed a dilated common bile duct (CBD) with filling defect suspicious of stone (Image). The patient subsequently underwent an endoscopic retrograde cholangio-pancreatography with removal of one stone and sphincterotomy. All symptoms improved, and the patient was discharged home with appropriate follow up.

## DISCUSSION

Approximately 5% of patients who have undergone cholecystectomy continue to have symptoms of abdominal pain, vomiting, dyspepsia, loose stool, and are thought to suffer from postcholecystectomy syndrome (PCS).^1^ The incidence of retained stone is as high as 10–15%.^2^ Patients with abnormal LFTs or an US showing a dilated CBD should be considered for a MRCP. It is the next appropriate step for patients with low to moderate risk of choledocolithiasis. Additionally, it is relatively non-invasive.^1^

CBD stones are a serious complication after cholecystectomy, therefore the diagnosis of PCS must always be considered in patients status post cholecystectomy with upper abdominal pain.^2^ MRCP is a useful but underused diagnostic tool and its routine use in the ED may significantly reduce morbidity and mortality.^2^ Although not a common diagnostic study in emergency medicine, it will likely become more prominent to avoid unnecessary admissions requiring emergency medicine providers to be more familiar with this tool.

**CPC-EM Capsule**

What do we already know about this clinical entity?

*Magnetic resonance cholangiopancreatography* (*MRCP) identifies retained stones in patients with prior gallbladder surgery, however MRCP is not a tool readily available to emergency physicians.*

What is the major impact of the image(s)?

*Given the wealth of information provided, perhaps MRCP imaging can be incorporated as part of a routine postcholecystectomy syndrome* (*PCS) workup and help to limit unnecessary hospital admissions.*

How might this improve emergency medicine practice?

MRCP imaging can help to improve diagnostic capabilities for patients suffering from PCS presenting to the emergency department.

## Figures and Tables

**Image:**
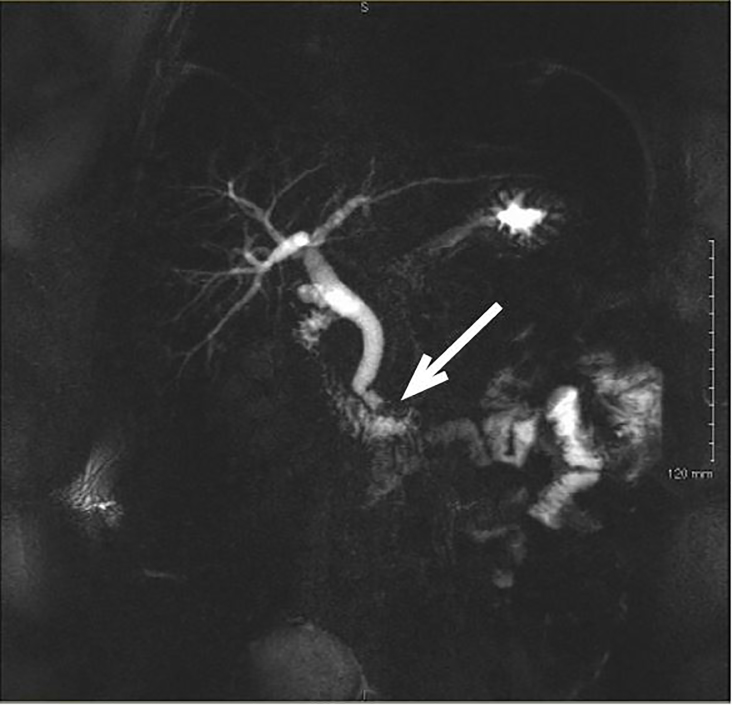
Magnetic resonance cholangiopancreatography illustrating a persistent distal filling defect (arrow) of the common bile duct near the ampulla of vater suspicious for retained stone with slightly dilated common bile duct.

## References

[1] Schofer JM (2010). Biliary causes of postcholecystectomy syndrome. J Emerg Med.

[2] Shapey IM, Jaunoo SS, Arachchilage KM (2012). Bilary tract imaging for retained calculi after laparoscopic cholecystectomy: is risk stratification useful?. Surg Laparosc Endosc Percutan Tech.

